# Calbindin-D32k Is Localized to a Subpopulation of Neurons in the Nervous System of the Sea Cucumber *Holothuria glaberrima* (Echinodermata)

**DOI:** 10.1371/journal.pone.0032689

**Published:** 2012-03-07

**Authors:** Carlos A. Díaz-Balzac, María I. Lázaro-Peña, Enrique M. García-Rivera, Carlos I. González, José E. García-Arrarás

**Affiliations:** 1 Department of Genetics, Albert Einstein College of Medicine, Bronx, New York, United States of America; 2 Department of Biology, University of Puerto Rico-Río Piedras Campus, Río Piedras, San Juan, Puerto Rico; University of South Florida College of Medicine, United States of America

## Abstract

Members of the calbindin subfamily serve as markers of subpopulations of neurons within the vertebrate nervous system. Although markers of these proteins are widely available and used, their application to invertebrate nervous systems has been very limited. In this study we investigated the presence and distribution of members of the calbindin subfamily in the sea cucumber *Holothuria glaberrima* (Selenka, 1867). Immunohistological experiments with antibodies made against rat calbindin 1, parvalbumin, and calbindin 2, showed that these antibodies labeled cells and fibers within the nervous system of *H. glaberrima*. Most of the cells and fibers were co-labeled with the neural-specific marker RN1, showing their neural specificity. These were distributed throughout all of the nervous structures, including the connective tissue plexi of the body wall and podia. Bioinformatics analyses of the possible antigen recognized by these markers showed that a calbindin 2-like protein present in the sea urchin *Strongylocentrotus purpuratus*, corresponded to the calbindin-D32k previously identified in other invertebrates. Western blots with anti-calbindin 1 and anti-parvalbumin showed that these markers recognized an antigen of approximately 32 kDa in homogenates of radial nerve cords of *H. glaberrima* and *Lytechinus variegatus*. Furthermore, immunoreactivity with anti-calbindin 1 and anti-parvalbumin was obtained to a fragment of calbindin-D32k of *H. glaberrima*. Our findings suggest that calbindin-D32k is present in invertebrates and its sequence is more similar to the vertebrate calbindin 2 than to calbindin 1. Thus, characterization of calbindin-D32k in echinoderms provides an important view of the evolution of this protein family and represents a valuable marker to study the nervous system of invertebrates.

## Introduction

EF-hand domain-containing calcium-binding proteins (CBP) of the calbindin and parvalbumin subfamilies have been used during the last three decades as markers of subpopulation of neurons in the nervous system of vertebrates [1]–[11]. Despite the vast interest in these proteins, their study in non-vertebrate species has not been widely pursued. The first major reports in non-vertebrates were conducted in *Loligo pealeii* (Lesueur, 1821) (Teuthida, Cephalopoda) [12] and *Drosophila melanogaster* (Meigen, 1830) (Diptera, Insecta) [13]. In the former a protein biochemically similar to calbindin 1 was characterized, though its sequence is not yet available. In the latter, the calbindin-D32k gene of *Drosophila* was cloned. This protein presents 42% and 37% homology to chicken calbindin 2 and calbindin 1, respectively [13]. Since then, sequences similar to calbindin-D32k have been identified in many representatives of Insecta as part of the whole genome sequencing being performed to study their evolution. So far, no other CBP of the calbindin or parvalbumin subfamilies has been reported to be present in a non-vertebrate organism.

The calbindin subfamily of EF-hand domain-containing CBP is characterized by having six EF-hand motifs and is made up by calbindin 1 (calbindin-D28k), calbindin 2 (calretinin), and secretagogin. These proteins function as calcium buffers, but their physiological role remains unknown [14], [15]. In vertebrates, specifically in mammals, calbindin 1, parvalbumin and calbindin 2 are present in different populations of neurons and interneurons of mice adult spinal cord [10]. In amphibians, differential immunoreactivity to anti-calbindin 1 and anti-calbindin 2 in cells and fibers throughout the brainstem serves to determine the localization and signature of many cell groups [9]. Lastly, calbindin 1 and calbindin 2-like immunoreactivities are present in the retina of the lamprey, the basal vertebrate studied at present [16]. In invertebrates, only two members of the calbindin subfamily have been identified, calbindin-D32k [13] and secretagogin [17]. In *Drosophila*, calbindin-D32k is selectively distributed in the nervous system, consistent with the specific distribution of the vertebrate calbindins [13]. Thus far, reports showing the distribution of any other calbindin subfamily members in invertebrates are lacking.

At present, the parvalbumin subfamily of EF-hand domain-containing CBP is one of the best-studied CBP subfamilies. These proteins have been well characterized at the molecular expression, and structural levels [18]–[22]. This subfamily is characterized by having two functional EF-hand motifs that bind calcium and is made up by two major isoforms, α and β (oncomodulin). In some organisms such as zebrafish, 9 isoforms, encoded by different genes, have been identified [19]. This protein is considered to be a vertebrate specific protein, as it has not been found in non-vertebrates. Interestingly, studies done with antibodies that recognize parvalbumin showed that there is parvalbumin-like immunoreactivity in the planarian nervous system [23].

Currently not much is known about the physiological function of CBP [24], but understanding their evolution may give us a good insight into their possible function. Thus identifying and studying CBP in basal deuterostomes, such as echinoderms, may be the key to unlocking this mystery. The phylum Echinodermata comprises a distinctive group of marine invertebrates characterized by radial pentameric symmetry originating in bilaterally symmetrical larvae. Echinoderms can be further subdivided into three subphylums, Asterozoa, Echinozoa, and Crinozoa. The subphylum Asterozoa includes the Asteroidea (starfishes) and Ophiuroidea (brittlestars); the subphylum Echinozoa includes the Echinoidea (sea urchins and sand dollars) and Holothuroidea (sea cucumbers); and the subphylum Crinozoa comprises the Crinoidea (sea lilies). These organisms are part of the branch of the deuterostome evolutionary tree that gave rise to chordates [25], thus, lie at a key evolutionary position. Therefore, studying CBP in echinoderms is vital in order to understand the evolution of these proteins in the chordates.

In this study we tested three antibodies that recognize EF-hand domain containing CBP of the calbindin and parvalbumin subfamilies. We identified a subpopulation of cells and fibers of the nervous system of *H. glaberrima* that showed immunoreactivity to these antibodies. Characterization of the antigens recognized by these markers was done by bioinformatics analyses with *S. purpuratus and H. glaberrima*, and western blot experiments with *H. glaberrima* and *L. variegatus*. Additionally, we used these antibodies, together with the novel neuronal marker RN1, to further characterize the nervous system component of the connective tissues in *H. glaberrima*. Our findings provide new markers for a subpopulation of neurons and fibers in the echinoderm nervous system. The availability of these new markers make feasible the study of the evolutionary aspects of the calcium-binding proteins family, as has been done recently with other gene family, the retinal genes in *S. purpuratus* [26]. Furthermore, we identified the presence of calbindin-D32k in *H. glaberrima* and *L. variegatus*, supporting its presence in at least two classes of echinoderms Our data also supports the evolutionary conservation of the calbindin subfamily, which seems to have evolved from two primitive EF-hand domain-containing CBP. One of them gave rise to cabindin-D32k in invertebrates and to calbindin 2 and calbindin 1 in vertebrates, while the other gave rise to secretagogin in both vertebrates and invertebrates. Furthermore, these proteins are preferentially distributed to the nervous system in both vertebrates and invertebrates.

## Materials and Methods

### Animals

Adult *Holothuria glaberrima* (Selenka, 1867) (Aspidochirotida, Holothuroidea) and adult *Lytechinus variegatus* (Lamarck, 1816) (Temnopleuroida, Echinoidea) specimens were collected from the shores of the north coast of Puerto Rico. The animals were kept in sea water aquaria at the University of Puerto Rico in Río Piedras.

### Tissue sections

*H. glaberrima* specimens were anesthetized in 0.2% 1,1,1-trichloro-2-methyl-2-propanol (Sigma, St. Louis, MO) for 10 min and dissected by longitudinal section of the body wall. Ventral and dorsal areas of the body wall were divided into anterior, middle and posterior portions, dissected and fixed in 4% paraformaldehyde at 4°C for approximately 1 h. Tissues were rinsed 3 times for 15 min with 0.1 M phosphate-buffered saline (PBS), and left in a 30% sucrose solution at 4°C. Once the tissues had been in 30% sucrose solution for at least 24 h, they were embedded in Tissue-Tek (Sakura Finetek, Torrance, CA). Cryostat tissue sections of 14 µm were cut and mounted on Poly-L-lysine-coated slides.

### Immunohistochemistry

The indirect immunofluorescence method was followed [27], [28]. In brief, tissues were rinsed for 5 min in 0.1 M PBS, followed by one rinse of 15 min in 1% Triton X, 1 h incubation with 0.1 M Glycine, and a 1 h incubation in goat serum 1∶50 (Invitrogen, Carlsbad, CA). Subsequently, the primary antibodies were incubated overnight at room temperature (Table 1). All antibodies were diluted in RIA buffer (0.05 M PBS- pH 7.4, 0.15 M NaCl, 0.5% BSA, and 1.5 mM NaN_3_). The primary antibodies used include the RN1 monoclonal antibody [27], [29] raised against a homogenate of the radial nerve of *H. glaberrima* and used at a dilution of 1∶100,000 in RIA buffer; the monoclonal antibody anti-β-tubulin, (Sigma T-4026 Lot. 024K4862) clone TUB 2.1 prepared against tubulin from rat brain and used at a 1∶500 dilution in RIA buffer; the rabbit antiserum anti-GFSKLYamide No. 23 2i2s [30] prepared against a GFSKLYa synthetic peptide and used at a 1∶1,000 dilution in RIA buffer; the rabbit antiserum anti-galanin-1 2i3s [31] prepared against galanin (Calbiochem Corp. San Diego, CA) and used at a 1∶1,000 dilution in RIA buffer; the rabbit polyclonal anti-calbindin 1 (Abcam ab11426 Lot. 378854) prepared against the calbindin 1 protein purified from rat kidney and used at a 1∶500 dilution in RIA buffer; the rabbit polyclonal anti-parvalbumin (Affinity Bioreagents PA1-933 Lot. 762-116) prepared against purified parvalbumin from rat skeletal muscle and diluted in RIA buffer to a 1 µg/ml concentration; the rabbit polyclonal anti-calbindin 2 (Abcam ab702 Lot. 721984) prepared against full length calbindin 2 and used at a 1/100 dilution in RIA buffer. Negative controls were performed in all experiments by incubating the tissue sections with rabbit serum during the incubation period of the primary antibody and following the normal immunostaining protocol.

**Table 1:** Antibodies used in this study.

*Antigen*	*Raised in*	*Immmunogen*	*Source*	*Dilution used*
Calbindin 1	rabbit (polyclonal)	28 kDa calbindin-D protein purified from rat kidney	Abcam (ab11426)	1∶500
Parvalbumin	rabbit (polyclonal)	Parvalbumin from rat skeletal muscle	Affinity Bioreagents(PA1-933)	1 µg/ml
Calbindin 2	rabbit (polyclonal)	Full length Calretinin	Abcam (ab702)	1∶100
GFSKLYamide	rabbit (polyclonal)	GFSKLYa synthetic peptide	Dr. García-Arrarás lab [30]	1∶1,000
Galanin	rabbit (polyclonal)	Galanin	Dr. García-Arrarás lab [31]	1∶1,000
β-tubulin	mouse (monoclonal)	Tubulin from rat brain	Sigma (T-4026)	1∶500
Unknown (RN1)	mouse (monoclonal)	Homogenate of the radial nerve of *H. glaberrima*	Dr. García-Arrarás lab [27]	1∶100,000
For more detailed information, see Materials and Methods.				

The secondary FITC antibodies, Goat anti-mouse (Biosource, Camarillo, CA, #AMI0408 Lot. 3501) and Goat anti-rabbit (Biosource, Camarillo, CA, #ALI0408 Lot. 1502) were used at a 1∶50 dilution in RIA buffer for double-labeling indirect immunohistochemistry. Also, the Cy3-conjugated secondary antibodies, Goat anti-mouse (Jackson ImmunoResearch Laboratories, Inc. West Grove, PA, #115-165-068 Lot. 47814) and Goat anti-rabbit (Jackson ImmunoResearch Laboratories, Inc. West Grove, PA, #111-165-144 Lot. 50694), were used at a 1∶2,000 dilution in RIA buffer for double-labeling indirect immunohistochemistry.

Cell nuclei were stained with 2 µM DAPI (Sigma, St. Louis, MO) in the buffered glycerol solution used to mount the slides. In cases where double-labeling was performed, the two primary antibodies were applied simultaneously and later the two secondary antibodies were added together (see [28]). Tissues were examined and photomicrographs taken on a Nikon Eclipse E600 fluorescent microscope with FITC, R/DII and DAPI filters. Images were recorded using the MetaVue software (version 6.0; MDS Analytical Technologies, Toronto, Canada) or the Spot Basic software (version 4.7; Diagnostic Instruments, Sterling Heights, MI), and Image J (version 1.37; NIH, Bethesda, MD). These were cropped, brightness and contrast adjusted, using Adobe Photoshop 7.0 (Adobe Systems, San Jose, CA).

### Bioinformatics

Sequences were downloaded from the NCBI GeneBank protein database and stored in Biology Workbench v3.2 [32]. BlastP were performed against the public nonredundant protein database in GeneBank. EF-hand domain determinations were performed using ScanProsite [33]. Alignments were carried out with ClustalW and edited with GeneDoc CLC free-workbench software (v.3.2.1 by CLC bio A/S, Denmark), or by using the Blosum62 matrix and edited with Geneious software (v.5.4.6 by Biomatters LTD, New Zealand) [34]. The phylogenetic trees were constructed using the unweighted pair group method with arithmetic mean (UPGMA) with default parameters and distances calculated using the Jukes-Cantor genetic distance model using the Geneious software (v.5.4.6 by Biomatters LTD, New Zealand) [34].

### Western blots

The western blot assay protocol used has been described previously [27]. Briefly, approximately 300 µg of radial nerve homogenates were run at 100 V in SDS-PAGE (5% stacking and 15% running gel) for 2 h using a BioRad Mini Protean Electrophoresis system. The gel was equilibrated for 10 min in Towin buffer and transferred to a PVDF membrane at 100 V for 1 h in a Mini Trans Blot Cell (BioRad, Hercules, CA). The membrane was then blocked for 1 h at room temperature with 5% nonfat dry milk and washed three times in Tris-buffered saline with 0.1% Tween-20.

The membrane was then incubated overnight with the respective primary antibody. The primary antibodies used were anti-calbindin 1 (1 µg/ml); anti-parvalbumin (1 µg/ml); anti-calbindin 2 (1∶100), diluted with RPMI 1640 medium supplemented with 5% horse serum. Positive controls in which mouse kidney homogenates were treated with the anti-calbindin 1 and the anti-calbindin 2 primary antibodies, and rat skeletal muscle homogenates with the anti-parvalbumin primary antibody were done to corroborate the quality of the homogenates and the antibody binding. Negative controls were done by using the EF-hand domain-containing synthetic protein EF-hand domain protein 2 (EFHD2) [35]. Following primary antibody incubation, the membrane was washed three times for 5 min each, and then incubated for 1 h in sheep anti-mouse (1∶10,000 dilution) or donkey anti-rabbit (1∶20,000 dilution) IgG peroxidase-linked secondary antibody (Amersham Biosciences, Piscataway, NJ) diluted with RPMI 1640 supplemented medium. After three washes of 5 min each, the membrane was incubated for 5 min in Super Signal West Dura Chemiluminescent Substrate (Thermo Fisher Scientific, Rockford, IL) and the signal was visualized using a Chemi System (UVP BioImaging Systems).

### Cloning of the *H. glaberrima* calbindin-D32k

cDNA used in the cloning was prepared from *H. glaberrima* radial nerve extracts. After dissection of the radial nerves, the tissues were placed in 1 ml of TRIzol Reagent (Invitrogen, Calsbad, CA) and homogenized with a PowerGen Model 125 Homogenizer (Thermo Fisher Scientific, Rockford, IL). Following a phenol-chloroform extraction, total RNA was obtained from the aqueous supernatant. cDNA was then synthesized from the total RNA using M-MLV Reverse Transcriptase (Invitrogen, Calsbad, CA) and oligo(dT) primers. Degenerate primers (F5′-GACCAGAATAAGGATGGAAAG-3′, R5′-ACTCTGCCCCGTGATTTT-3′) were designed against a potential calbindin 2 homolog found in the *S. purpuratus* genome (XP_001178717) and used to amplify a 376 bp fragment from *H. glaberrima* radial nerve cDNA. A highly conserved region was chosen for amplification by aligning sea urchin calbindin 2-like protein (XP_001178717) with calbindin-D32k from *D. melanogaster* (NP_476838) and calbindin 2 from *M. musculus* (NP_033918). A start and stop codon were affixed to the 5′ and 3′ positions of the insert, respectively, using PCR amplification (F5′-ATGGACCAGAATAAGGATGGAAAG-3′, R5′-TCAACTCTGCCCCGTGATTTT-3′). Using this template, the restriction sites BamHI and NotI were affixed to the 5′ and 3′ positions, respectively, using PCR (F5′-GAAATA**GGATCC**ATGGAC CAGAATAAGGATGGAAAG-3′, R5′-TTCCCC**GCGGCCGC**TCAACTCTGCCC CGTGATTTT-3′). This product was purified using a QIAGEN Spin Column (QIAGEN, Valencia, CA) and inserted into pGEX6P-1 (GE Healthcare Biosciences, Piscataway, NJ), using BamH1 and NotI restriction enzymes (New England Biolabs, Ipswich, MA). The vector was previously treated with Calf Intestinal Alkaline Phosphatase (Invitrogen, Carlsbad, CA) diluted to 0.01 u/uL in Buffer CIAP. The ligation was carried out using T4 ligase (New England Biolabs, Ipswich, MA), and the construct was heat shocked into OneShot BL21 Star (DE3) chemically competent *E. coli* cells (Invitrogen, Carlsbad, CA). After the shock, vials were incubated in SOC medium (Invitrogen, Calsbad, CA) for 1 h before plating. We analyzed for positive insertion using selective plating with ampicillin and Colony PCR, using pGEX specific primers. All DNA samples were analyzed in 1% agarose gels run at 150 V for 30 min in 10 mM Sodium Borate buffer, visualized with SafeView Classic (Applied Biological Materials, Richmond, BC) staining in a ChemiDoc XRS+ System, and Sanger sequencing was use to confirm the sequences.

### Expression of *H. glaberrima* calbindin-D32k

Positive colonies were grown in LB medium with 50 µg/ml ampicillin to O.D._600_ 1.3, and induced with 1 mM IPTG (Sigma, St. Louis, MO). Cells were lysed using B-PER Reagent (Thermo Fisher Scientific, Rockford, IL) and protein extraction was carried out using TRIzol LS Reagent (Invitrogen, Calsbad, CA) according to the manufacturers protocol for bacterial protein extraction. The fragment was purified using a B-PER GST Fusion Protein Spin Purification Kit (Pierce, Rockford, IL), and fractions were collected after lysing, washing, and elution, for a total of seven fractions. As a negative control, another protein from a different bacterial strain containing the same vector but a different insert, translationally controlled tumor protein (TCTP), was purified along with our fragment. The fractions were then analyzed via SDS-PAGE and western blot.

## Results

### Neuronal specificity

**Figure 1:**
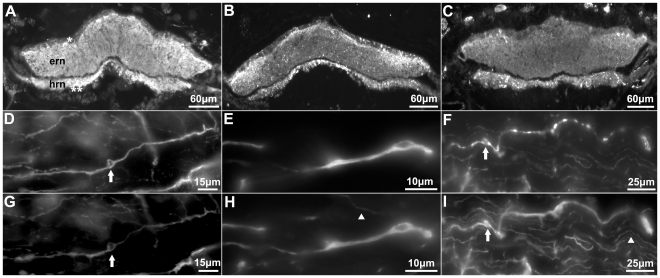
Anti-calbindin 1, anti-parvalbumin, and anti-calbindin 2 immunoreactivity in the nervous system of *Holothuria glaberrima* (Holothuroidea). Transverse sections through the radial nerve cords showing immunoreactivity to anti-calbindin 1 (A), anti-parvalbumin (B), and anti-calbindin 2 (C) in the ectoneural and hyponeural components of the radial nerve. Double-labeling of anti-calbindin 1 (D) and RN1 (G), anti-parvalbumin (E) and RN1 (H), and anti-calbindin 2 (F) and RN1 (I) in cells (arrows) and fibers of the connective tissue plexus of the tube feet. Co-labeling is observed with the anti-CBPs markers and RN1 in the majority of the fibers, but not in all (arrowheads). ern, ectoneural component of the radial nerve cord; hrn, hyponeural component of the radial nerve cord; *, ectoneural component cell bodies; **, hyponeural component cell bodies.

The radial nerve cord (RNC) is the most prominent neural structure of the echinoderm's nervous system. It is subdivided anatomically into two major components, the ectoneural (ERN) and the hyponeural component (HRN) (Fig. 1A). The RNC is a ganglionated nerve, with cell bodies located in the periphery of the respective component. Nerves originating from the RNC innervate other body structures. The ectoneural peripheral nerve extends from the ERN into the dermis and tube feet, while the hyponeural peripheral nerve extends from the HRN into the body wall travelling between the coelomic epithelia and the circular muscle. We observed immunoreactivity to anti-calbindin 1, anti-parvalbumin, and anti-calbindin 2 within both components of the RNC (Fig. 1A–C). All three antibodies labeled cells in the RNC, but their numbers were not the same. Approximately 30±4 (6%) (n = 7) anti-calbindin 1 immunopositive cells were observed per section [19±4 (4%) in the ERN and 11±4 (2%) in the HRN], as demonstrated by double-labeling with DAPI. A smaller number of cells were labeled with anti-parvalbumin [14±4 (3%) (n = 4) cells per section- 9±3 (2%) in the ERN and 5±2 (1%) in the HRN] and with anti-calbindin 2 [4±1 (n = 4) (1%) cells per section- 4±1 (1%) in the ERN and 0 (0%) in the HRN] (all numbers are expressed as mean ± SD).

Anti-calbindin 1 immunoreactivity was observed in a large number of fibers equally distributed throughout both components of the RNC, contrary to fibers immunoreactive to anti-parvalbumin and anti-calbindin 2, which were mostly distributed to the distal region of the ERN and all throughout the HRN. Immunoreactivity to the three markers was also observed in the peripheral nerves extending from the ERN and the HRN. Individual immunoreactive fibers were best observed within the circular muscle, just distal to the hyponeural peripheral nerve. At this site, fibers immunoreactive to the anti-CBPs markers were present, but their number and morphology was different with each marker as observed in serial sections. The number of immunoreactive fibers was larger with anti-calbindin 1 and lowest with anti-calbindin 2, leaving the amount of anti-parvalbumin immunopositive fibers in-between the first two. The thickness of immunoreactive fibers also differed in the same pattern. Anti-calbindin 1 labeled thick and thin fibers, while most of anti-calbindin 2 immunopositive fibers were thick fibers. Anti-parvalbumin immunopositive fibers were mostly thick fibers, though some thin fibers were observed. The localization of the fibers, as well as the pattern of direction in which the fibers run through was similar with all the three markers.

**Figure 2:**
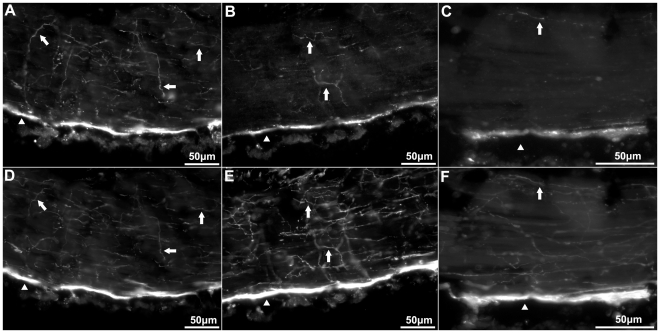
Anti-calbindin 1, anti-parvalbumin, and anti-calbindin 2 immunoreactivity in the peripheral nerve and circular muscle of *Holothuria glaberrima* (Holothuroidea). Longitudinal sections through the body wall showing colabeling of anti-calbindin 1 (A) and RN1 (D), anti-parvalbumin (B) and RN1 (E), and anti-calbindin 2 (C) and RN1 (F). Most of the immunoreactivity was observed in the peripheral nerves (arrowheads), while only a minor supopulations of fibers were co-labeled by the anti-CBP and RN1 (arrows).

We corroborated the neuronal specificity of the three markers by double-labeling immunohistochemistry using the CBP marker with the monoclonal antibodies RN1 or anti-β-tubulin, and by means of serial sections immunostained with markers that recognize the well-characterized neuropeptides GFSKLYamide and Galanin. Co-labeling of individual fibers with anti-calbindin 1 and RN1, anti-parvalbumin and RN1, and anti-calbindin 2 and RN1 was best observed in the circular muscle (CM) (Fig. 2). Although, most of the fibers were co-labeled, there were still fibers (especially those that had a smaller diameter) only labeled by RN1, but not by any of the CBPs markers.

### Connective tissue components

**Figure 3:**
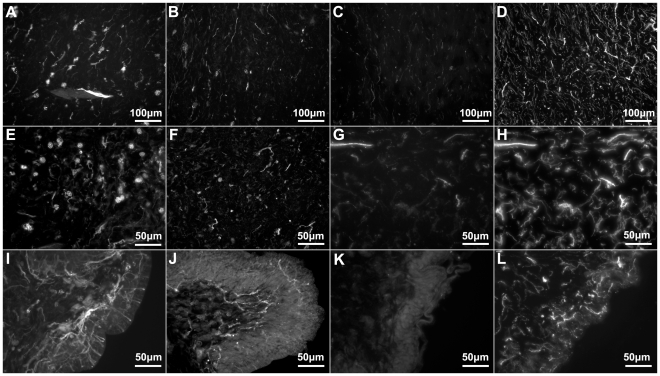
Anti-calbindin 1, anti-parvalbumin, and anti-calbindin 2 immunoreactivity in the body wall nervous system of *Holothuria glaberrima* (Holothuroidea). Longitudinal sections through the body wall showing immunoreactivity to anti-calbindin 1 (A,E,I), anti-parvalbumin (B,F,J), and anti-calbindin 2 (C,G,K) as compared to RN1 (D,H,L) immunoreactivity in the different layers of the body wall. Immunoreactivity of the internal connective tissue plexus (A–D), external connective tissue (E–H) and epidermis (I–L) showed that the anti-CBP only labeled a minor subpopulation of fibers and cells within these plexi, as compared to RN1 which labeled a large subpopulations of cells and fibers of the body wall nervous system.

The connective tissue component (CTC) of holothurians has been defined to be sandwiched between two epithelia and has been well described in the body wall and in the tube feet. This component is innervated by fibers that originate from the ectoneural nervous system. The CTC of the body wall can be divided into inner and outer dermis, according to the distribution of its fibers. All three markers labeled structures in the body wall CTC divisions, although more fibers were labeled in the outer dermis than in the inner dermis (Fig. 3). Both individual fibers and some small bundles were observed. Immunoreactive fibers in the inner dermis had a parallel orientation to the muscular fibers of the CM, while immunoreactive fibers in the outer dermis did not show a particular orientation (Fig. 3). In the inner dermis, fibers were oriented parallel to the circular muscle fibers that lie beneath. Cells immunoreactive to all three markers were also observed in the two dermal layers of the body wall. These cells were mostly isolated and had a bipolar or pyramidal morphology. Fibers could be observed extending from them through the dermis; though no association with a particular structure or localization could be identified.

**Figure 4:**
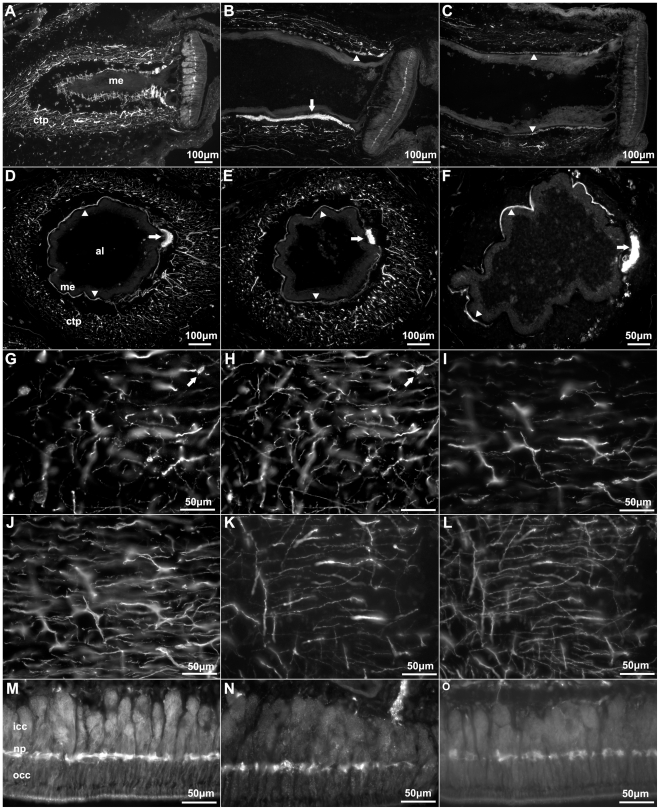
Anti-calbindin 1, anti-parvalbumin, and anti-calbindin 2 immunoreactivity in the podial nervous system of *Holothuria glaberrima* (Holothuroidea). Longitudinal and transverse sections through the tube feet showing immunoreactivity to anti-calbindin 1 (A,D,G,M), anti-parvalbumin (B,E,I,N), and anti-calbindin 2 (C,F,K,O) in the different subdivisions of the podial nervous system. (A–F) Immunoreactivity was observed with all markers in the podial nerve (arrows) and in the podial cylindrical fenestrated sheath (arrowheads). (G–L) Co-labeling of anti-calbindin 1 (G) and RN1 (H), anti-parvalbumin (I) and RN1 (J), and anti-calbindin 2 (K) and RN1 (L) was observed in fibers and cells (arrows) of the connective tissue plexus. (M–O) Anti-calbindin 1 (M), anti-parvalbumin (N), and anti-calbindin 2 (O) immunoreactivity was also present in the nerve plate of the tube feet's disk. Al, ambulacral lumen; ctp, connective tissue plexus; icc, inner cluster of cells; me, mesothelium; np, nerve plate; occ, outer cluster of cells.

Although the tube feet are extensions of the body wall, their CTC is somewhat different from the body wall dermis. A larger number of fibers within the CTC of the tube feet were labeled by all three markers, compared to the outer dermis. Thicker fibers were also noted to be present in the CTC of the tube feet compared to the body wall (Fig. 4). The fibers present within the CTC of the tube feet were closely packed and presented an orthogonal orientation that clearly differed from those of the body wall. Thus, they outlined a cylindrical shape that distinctly delineated the boundary between the tube feet and the body wall dermis. Immunoreactive cells to all three markers were observed in CTC of the tube feet (Fig. 1D–F). Similar to those cells within the body wall, these had a bipolar or pyramidal morphology, and fibers from these could in some cases be observed extending to the podial nerve and the podial cylindrical fenestrated sheath of the tube feet.

### EF-Hand comain containing CBPs

An alternative strategy to study the presence and relationship of EF-hand domain containing CBPs is to focus on the gene sequences identified in EST and genomic databanks. Sequences for EF-hand domain containing CBPs calbindin 1, calbindin 2, and parvalbumin in Echinodermata, were searched in the NCBI's GeneBank database (Echinodermata- TaxID: 7586), SpBase (the sea urchin *Strongylocentrotus purpuratus* genome annotation database), the *S. purpuratus* genome project [36], and the sea cucumber *H. glaberrima* expressed sequences tags EST database from our laboratory [37]. According to these databases only a putative calbindin 2 protein was found in the genome of *S. purpuratus*. Interestingly, no echinoderm sequences annotated for calbindin 1 or parvalbumin were found. However, we did find a secretagogin, which belongs to the calbindin subfamily of CBPs. To further characterize the most likely antigens being recognized by these antibodies in the echinoderm nervous system, we performed the following analyses: (1) an orthologous search for the respective protein sequence from *Rattus norvegicus* using the NCBI's GeneBank database (Echinodermata; TaxID: 7586) and the *S. purpuratus* annotated genome in SpBase and (2) structural comparison of the EF-hand domains of related proteins. If an orthologue sequence was obtained, multiple sequences alignments were performed.

### Calbindin 1 or calbindin-D28k

**Figure 5:**
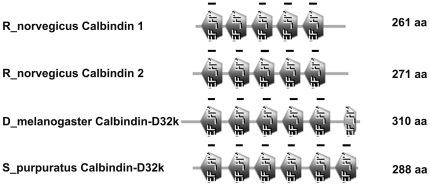
Schematic domain structure of members of the calbindin subfamily in rat (*R. norvegicus*), fly (*D. melanogaster*), and sea urchin (*S. purpuratus*). Domains 1, 3, 4, and 5 are present in all the proteins. The second domain doesn't bind calcium in the calbindin 1 protein, while it does in calbindin 2, and in *Drosophila and S. purpuratus'* calbindin-D32k. Domain 6 is present in *Drosophila* calbindin-D32k and *S. purpuratus* calbindin-D32k, but it is only able to bind calcium in the latter. Pairwise similarities of domains between proteins were calculated by aligning the domains as predicted by ScanProsite.

A BLAST of rat calbindin 1 (AAH81764.1) against the NCBI Echinodermata sequences was performed to identify the presence of orthologous proteins in the echinoderms. Rat calbindin 1 sequence was used since this was the immunogen against which the anti-calbindin 1 antibody was made. The best hit obtained in this search was a predicted protein known as “similar to ENSANGP00000013966 isoform 2” (XP_781517.2), which corresponded to the *S. purpuratus* putative calbindin 2, as annotated by Kathy Foltz [17], with an E value of 1e^−50^ and a Score of 196 (from now on CalretIso2 or Sp_Calb32). The second best hit was the isoform 1 of the same predicted protein (XP_001178717.1) (from now on CalretIso1) with an E value of 4e^−50^ and a Score of 194; while the third best hit was a hypothetical protein (XP_785060.2), which corresponds to the *S. purpuratus* secretagogin, as annotated by Kathy Foltz [17], (from now on Sp_SCGN), with an E value of 3e^−31^ and a Score of 131. A comparison performed between both calbindin 2 isoforms identified in *S. purpuratus* sequences revealed two slight differences between them. First, isoform 1 had nine more amino acids than isoform 2 (residues 204 to 212); and second, they differed in five amino acids (residues 198 to 203). An alignment between calretIso2 from *S. purpuratus* and calbindin 1 from *R. norvegicus* showed 42% of identities and 60% of positives. Nonetheless, these two sequences differed in length and molecular weight. In rats, like in other vertebrates, calbindin 1 has 261 amino acids with a molecular weight of 28 kDa. Meanwhile, the sea urchin calretIso2 had 288 amino acids with a predicted molecular weight of 32 kDa. Structural comparative analysis performed between rat calbindin 1, rat calbindin 2, *Drosophila* calbindin-D32k and the sea urchin calbindin-D32k sequences using the ScanProsite program showed that all proteins had different numbers of EF-hand domains. Rat calbindin 1 and 2 had only 5 EF-hand domains, of which 4 and 5 of these are capable of binding Ca^2+^ respectively, while *Drosophila* and the *S. purpuratus* calbindin-D32k had 6, of which 5 and 6 of these are capable of binding Ca^2+^ respectively (Fig. 5).

Furthermore, a BLAST of calretIso2 against GeneBank showed that the most similar sequence was a predicted protein similar to calbindin-D32k (XP_002735965.1) of the hemichordate *Saccoglossus kowalevskii* (Agassiz, 1873) (Enteropneusta, Hemichordata). The second best hit was another predicted protein similar to calbindin-D32k (XP_975683.1) from the invertebrate *Tribolium castaneum* (Herbst, 1797) (Insecta, Arthropoda). A multiple alignment of the best hits of calbindin 1 sequences (-D28k and -D32k) with 11 species (5 from vertebrates and 4 from invertebrate), suggested that calretIso2 might correspond to calbindin-D32k in the sea urchin. A phylogenetic tree from this analysis showed that Sp_Calb32 clustered with the calbindin-D32k. All together, these results suggested that the calbindin 2-like protein annotated in the genome of *S. purpuratus* is the echinoderm orthologue of calbindin-D32k.

### Calbindin 2 or calretinin

The BLAST search for calbindin 2 using *R. norvegicus* protein sequence (NP_446440.1), against the NCBI GeneBank database (Echinodermata- TaxID: 7586) showed that the most similar hits were the same as for calbindin 1, though with lower E values and higher scores: calretIso2 had an E value of 1e^−57^ and a Score of 218, calretIso1 had an E value of 1e^−56^ and a Score of 214, and the Sp_SCGN had an E value of 1e^−36^ and a Score of 147. Structural comparative analysis performed between rat calbindin 2 and the sea urchin calretIso2 sequences using the ScanProsite program showed that both proteins are mainly composed of EF-hand domains, but rat calbindin 2 had only 5 domains, all of which were capable of binding Ca^2+^, while the sea urchin calretIso2 had 6 all of which were capable of binding Ca^2+^ (Fig. 5). A multiple alignment of calbindin 2 sequences, calbindin-D32k, and the calretIso2 also suggested that the latter might correspond to calbindin-D32k in the sea urchin. A phylogenetic tree from this analysis showed that calretIso2 was clustered with calbindin-D32k. These results also support our contention that the calbindin 2-like protein annotated in the genome of *S. purpuratus* is the echinoderm orthologue of calbindin-D32k.

### Calbindin-D32k

**Figure 6:**
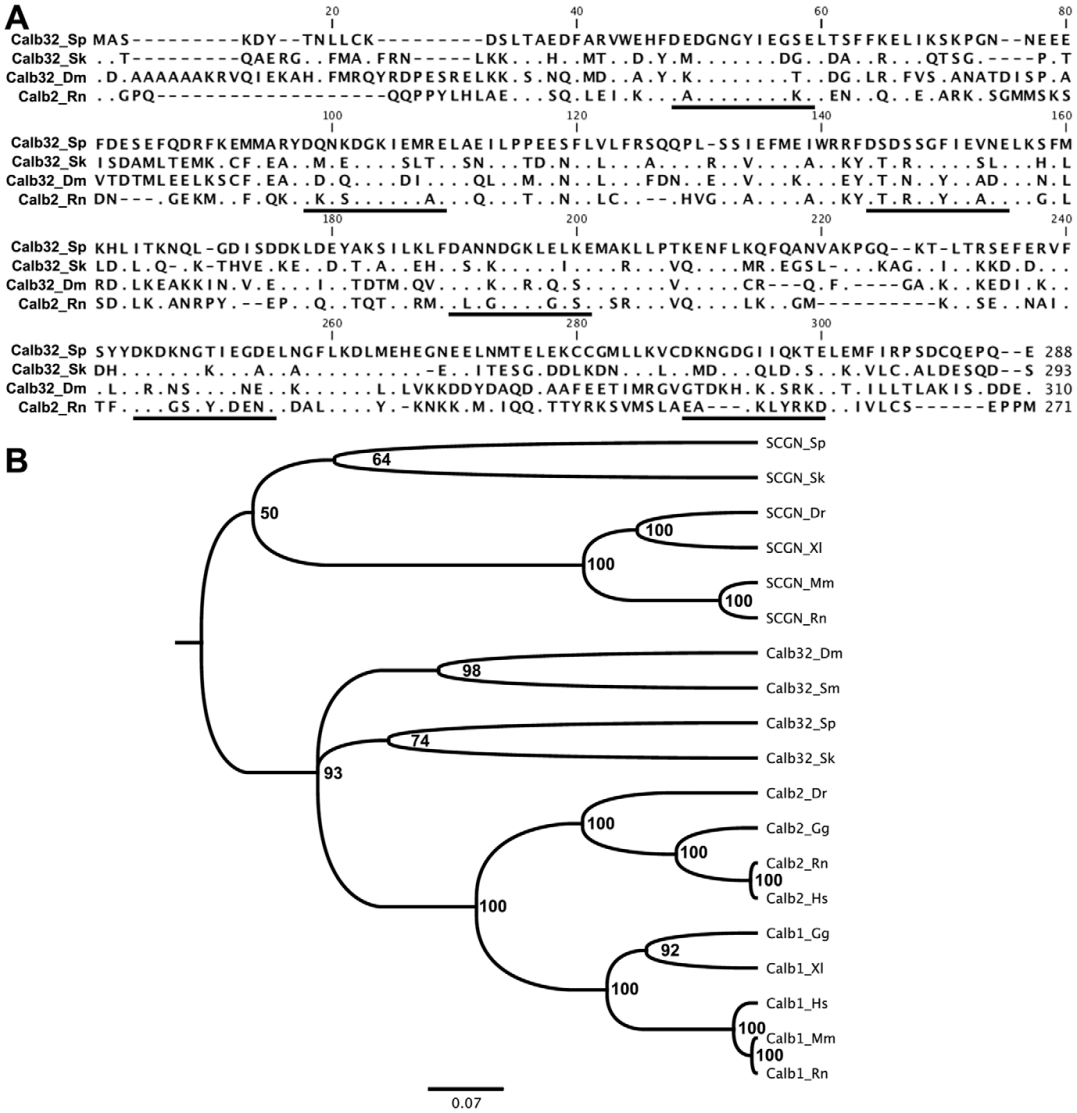
Alignment of the peptide sequences and phylogenetic trees of members of the calbindin subfamily from different species. (A) Sequence alignment of rat calbindin 2 (NP_446440.1), *Drosophila* calbindin-D32k (NP_476838.1), *S. kowalevskii* calbindin-D32k (XP_002735965.1), and *S. purpuratus* calbindin-D32k proteins (XP_781517.2). Alignment of the proteins was made using the CLUSTALW multiple sequences alignment. Dots represent conserved residues and black lines below the residues represents the presence of an EF hand domain. (B) Phylogenetic tree of members of the calbindin subfamily from different species. Calb32_Dm *Drosophila melanogaster* (NP_476838.1), Calb32_Sk *S. kowalevskii* (XP_002735965.1), Calb32_Sp *S. purpuratus* (XP_781517.2), Calb32_Sm *S. mansoni* (XP_002574332.1), Calb2_Gg *G. gallus* (NP_990647.1), Calb2_Dr *D. rerio* (NP_957005.1), Calb2_Rn *R. norvegicus* (NP_446440.1), Calb2_Hs *H. sapiens* (NP_001731.2), Calb1_Xl *X. laevis* (NP_00108408.1), Calb1_Gg G. gallus (NP_990844.1), Calb1_Mm *M. musculus* (AAH16421.1), Calb1_Rn *R. norvegicus* (AAH81764.1), Calb1_Hs *H. sapiens* (NP_004920.1), SCGN_Sk *S. kowalevskii* (NP_001161653.1), SCGN_Sp *S. purpuratus* (XP_785060.2), SCGN_Dr *D. rerio* (NP_001005776.1), SCGN_Xl *X. laevis* (NP_001088097.1), SCGN_Rn *R. norvegicus* (NP_963855.1), SCGN_Mm *M. musculus* (NP_663374.1). The phylogenetic tree was constructed from an alignment created by using the unweighted pair group method with arithmetic mean. The scale bar represents 100 times the expected number of amino acid substitution.

The invertebrate sequence most similar to the calretIso2 protein was that of calbindin-D32k from *S. kowalevskii*. A Blast of calretIso2 showed that the E value and score for this protein were 2e^−84^ and 261. Sequence alignment between these two proteins showed that they are 52% identical and 71% similar when conserved residues were included. As expected, the calbindin-D32k had a similar number of amino acids, when compared to calretIso2, 293 versus 282 amino acids, respectively. Structural analysis using the ScanProsite program showed that this protein had 6 EF-hand domains, all of which were capable of binding Ca^2+^ (Fig. 5). A multiple alignment of calbindin-D32k sequence in invertebrates, rat calbindin 2, and the *S. purpuratus* calbindin-D32k supported previous results showing a high similarity between calbindin 2 and the invertebrate calbindin-D32k, (Fig. 6a). In a phylogenetic tree done from a multiple alignment of representatives of the calbindin family (calbindin 1, calbindin 2, calbindin-D32k, and secretagogin), two major groups were formed (Fig. 6b). The first included calbindin 1, calbindin 2, and calbindin-D32k, and the second was composed of secretagogin. Within the first cluster, two major groups were derived, one containing calbindin-D32k and a second containing calbindin 1 and 2. The sea urchin calretIso2 was grouped with the calbindin-D32k as was expected from our previous analyses. Hence, from now on we will refer to calretIso2 as *S. purpuratus*' calbindin-D32k.

### Parvalbumin

The BLAST search for parvalbumin (using *R. norvegicus* protein sequence- NP_071944.1- against the NCBI GeneBank database -Echinodermata: taxid: 7586) showed a predicted protein similar to calmodulin (XP_001190813.1) from the sea urchin *S. purpuratus* to be the most similar match. Although the e-value seems to be relatively low (3e^−07^ and a score of 49.7), both sequences shared 33% of identities and 55% of positive residues. The structural analyses using ScanProsite between rat parvalbumin and the sea urchin predicted protein-similar to calmodulin showed that the first had two EF-hand domains, while the latter had four EF-hand domains. Interestingly, this predicted protein from sea urchin had 283 amino acids, while the rat parvalbumin had 110 amino acids. In the *S. purpuratus* genome database (Sea Urchin Genome Sequence Consortium 2006), three calmodulin sequences have been reported (GLEAN3_05032, GLEAN3_05033 and GLEAN3_16371). Moreover, among the higher hits from *R. norvegicus* Parvalbumin BLAST was calmodulin (AAY41437) from the Japanese sea cucumber *Apostichopus japonicus* (149 amino acids). Sequence alignment between these two proteins showed that they are 29% identical and 51% similar when positive residues were included. In summary, no sequence orthologous to the vertebrate parvalbumin was found in the echinoderms.

### Western blots

**Figure 7:**
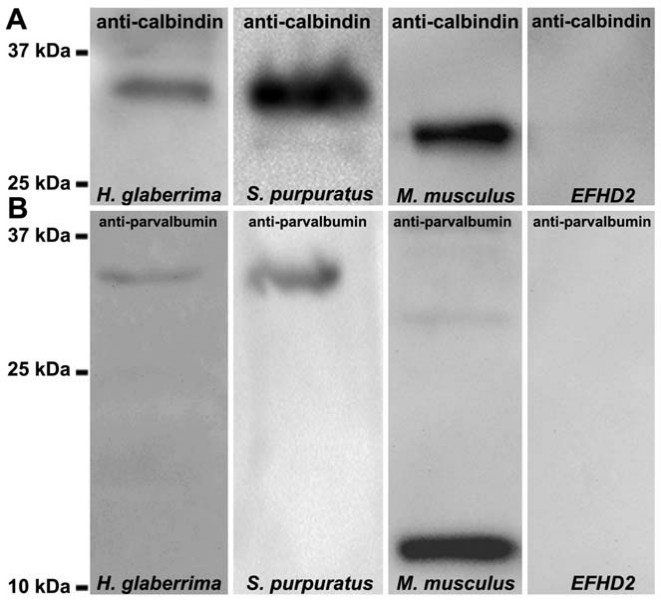
Western blot of holothurian and echinoid radial nerve cords preparations using anti-calbindin 1 and anti-parvalbumin. (A) A 32 kDa immunoreactive band to anti-calbindin 1 was observed in *H. glaberrima* and *L. variegatus* radial nerve cords homogenates. A 28 kDa immunoreactive band corresponding to calbindin 1 was observed in the rat kidney positive control and no immunoreactive bands were observed in the synthetic peptide of an EF-hand domain-containing protein negative control. (B) A 32 kDa immunoreactive band to anti-calbindin 1 was observed in *H. glaberrima* and *L. variegatus* radial nerve cords homogenates. A 12 kDa major immunoreactive band corresponding to calbindin 1 was observed in the rat skeletal muscle positive control and no immunoreactive bands were observed in the synthetic peptide of an EF-hand domain-containing protein negative control.

Immunoblots of nerve cord homogenates, from the sea cucumber *H. glaberrima* and the sea urchin *L. variegatus*, revealed a single immunoreactive band of approximately 32-kDa to the polyclonal anti-calbindin 1. Positive controls of mouse kidney homogenates showed a major immunoreactive band of 28-kDa (Fig. 7a). Interestingly, the immunoreactive band in a western blot of nerve cord homogenates performed with the polyclonal anti-parvalbumin, was similar to the one observed in the anti-calbindin 1 western blot; while in positive controls, using mouse skeletal muscle homogenates, a main band of approximately 12-kDa was observed (Fig. 7b). No immunoreactive band in nerve cord homogenates was observed in western blots done with the polyclonal anti-calbindin 2. Nonetheless, this antibody did not react either with homogenates of mouse kidney (the tissue from which the antigen was obtained) indicating that the antibody does not work in western blot. No bands were observed in the negative control (EFHD2 synthetic protein) demonstrating the specificity of the antigens recognized by these antibodies.

**Figure 8:**
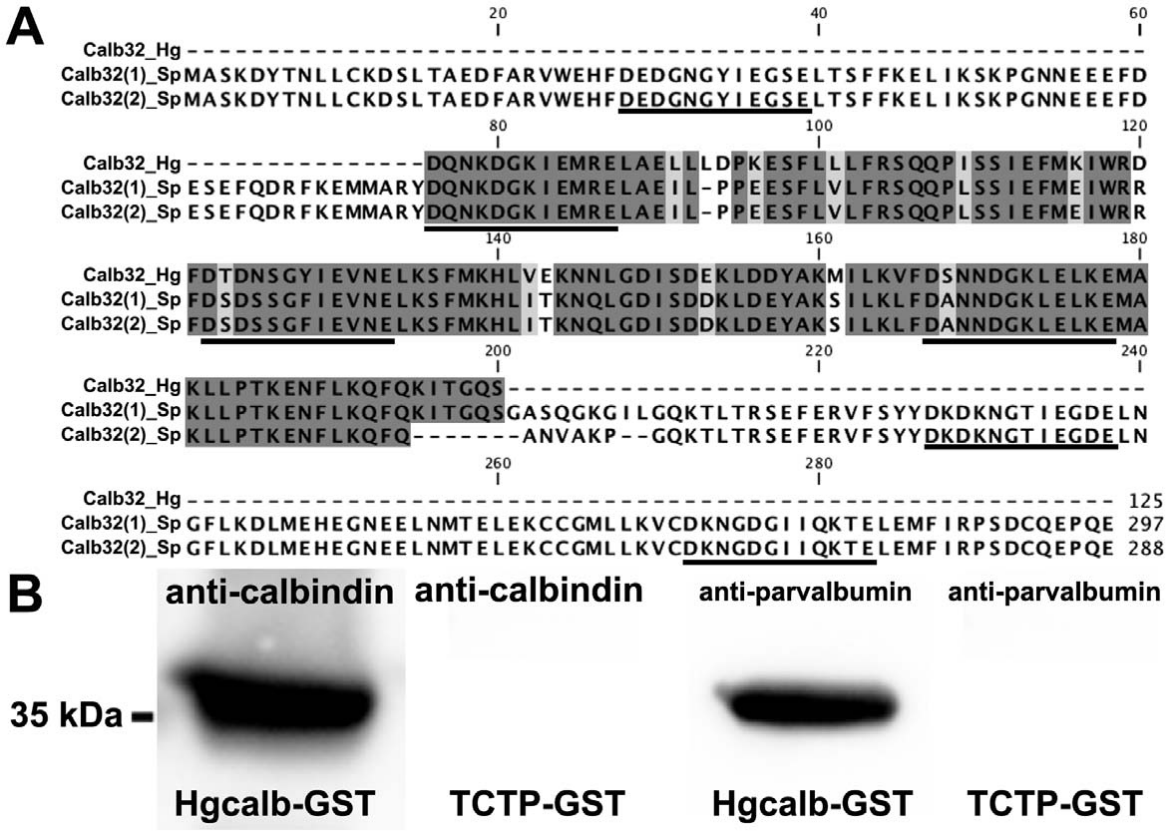
Identification of calbindin-D32k as the anti-calbindin 1 and anti-parvalbumin epitope in echinoderms. (A) Sequence alignment of *H. glaberrima* calbindin-D32k fragment (Calb32_Hg) and *S. purpuratus* calbindin-D32k isoform 1 (Calb32(1)_Sp) and isoform 2 (Calb32(2)_Sp), showing a high degree of homology. Alignment of the proteins was made using the CLUSTALW multiple sequences alignment. Dark gray represent conserved residues, light gray represent conservation of strong groups, and black lines below the residues represents the presence of an EF hand domain. (B) A 36 kDa immunoreactive band to anti-calbindin 1 and anti-parvalbumin was observed in the *H. glaberrima* GST-tagged calbindin-D32k fragment peptide (Calb32_Hg), and no immunoreactive band to anti-calbindin 1 and anti-parvalbumin was observed in the negative control, GST-tagged translationally controlled tumor protein (TCTP).

The previous results prompted us to search for the *H. glaberrima* ortholog of the predicted calbindin-D32k identified in echinoderms (XM_001177659.1). Using primers designed against a well-conserved region between *S. purpuratus* calbindin-D32k (XM_001177659.1), *Drosophila melanogaster* calbindin-D32k (NM_057490.3) and *Rattus norvegicus* calbindin 2 (NM_053988.1), we amplified a fragment of the *H. glaberrima* ortholog. The amplicon was a 376 bp region of the *H. glaberrima* calbindin-D32k homolog, (Hg_Calb32) containing 3 out of the 6 EF-hand domains identified in *S. purpuratus* calbindin-D32k (Fig. 8a). A homology search for the peptide sequence, resulted in the top hits being calbindin-D32k sequences in invertebrates, followed by calbindin 1 and calbindin 2 sequences in vertebrates. The top two hits were the two isoforms of calbindin-D32k of *S. purpuratus* with e values of 2e-68 (isoform 1) and 9e-64 (isoform 2), confirming that we had successfully identified a fragment of *H. glaberrima*'s calbindin-D32k. Even though the cloned fragment was almost identical to isoform 1 of *S. purpuratus*, it still holds some interesting differences. The main difference between isoform 1 and isoform 2 from the sea urchin is a 15 amino acid long sequence from 195 to 210, which is the region we selected for the reverse degenerate primer. As can be seen from the alignment of the isoforms with Hg_Calb32, our fragment corresponded to the equivalent of isoform 1 as it has the beginning of the 15aa region. Interestingly, this is a very variable region amongst the different calbindin sequences, and only isoform 1 seems to have this 15 amino acid motif. There are 6 variable residues between sea cucumber and sea urchin sequence. Of these, the addition of an amino acid in Hg_Calb32 at position 93 or 94 absent in the sea urchin sequences stands out, as it is not present in any of the other sequences.

Hg_Calb32 was cloned and expressed in an heterologous system. As our construct contained a GST tag, we employed Affinity Chromatography for the purification of the Hg_Calb32, which was then analyzed through SDS-PAGE. Coomassie staining showed enrichment of a ∼36 kDa band, which is near the expected weight of our fragment tagged with GST. A western blot of the expressed peptide tagged with GST was immunopositive to both anti-calbindin 1 and anti-parvalbumin, in the expected band size of 36 kDa (Fig. 8b), as well as when proved with an anti-GST antibody. No immunoreactive band was observed in the negative control, which consisted of GST tagged TCTP when incubated with anti-calbindin 1 or anti-parvalbumin (Fig. 8b), which was immunopositive when proved with the anti-GST antibody.

## Discussion

In this study we report the distribution of an EF-hand domain containing CBP of the calbindin subfamily in the nervous system of the echinoderms. This is the first report that describes the distribution of these proteins, which have been well-studied in the vertebrate nervous system, in the echinoderm nervous system. Accordingly, calbindin-D32k is specifically localized within the nervous system of the echinoderm *H. glaberrima*, extending the usefulness of this neural marker to invertebrates, in particular to the Echinodermata.

### Anti-calbindin 1, anti-parvalbumin, and anti-calbindin 2 immunoreactivity is restricted to the nervous system

The immunoreactivity of these CBP markers is specific to echinoderm neural components. The evidence for this is the following: First, in other organisms in which these have been studied (mostly mammals), these markers specifically label neural structures [9], [10], [16]. Second, in the holothurian studied here, immunoreactivity to these markers is observed in cells and fibers of the radial nerve cords, podial nerves and peripheral nerves, which are the classically described neural structures in the echinoderms [27], [29], [30], [38]–[40]. Finally, double-labeling experiments with known neural markers (RN1 and anti-β-tubulin), and serial sections done with markers made against the neuropeptides GFSKLYamide and Galanin show co-labeling of the same fibers and cells by the CBP markers and the neural markers. Similarly, the localization of some of the fibers and cells labeled by anti-GFSKLYamide and anti-Galanin is similar to that of the three CBP markers in serial sections.

### Anti-CBP immunoreactivity identified distinctive plexi within the connective tissue

One of the most interesting findings of our study is the immunoreactivity of the CBP markers in the connective tissue plexi of the body wall and tube feet. The only other marker that has been used to identify these plexi is RN1 [27], [29]. The fact that the CBP markers recognize these plexi is very interesting from a functional point of view. Trotter and Koob [41] demonstrated that calcium-dependent processes are involved in the stiffening of the body wall in the sea cucumber *Cucumaria frondosa* (Gunnerus, 1770) (Holothuroidea, Dendrochirotida). The importance of calcium in the connective tissue catch phenomenon has also been demonstrated due to its association with the electron-dense granules in the juxtaligamental cells [42] and its role in the hardening and softening of the intervertebral ligament of ophiruoids [43]. Therefore, the echinoderms EF-hand containing CBPs are excellent candidates to act as calcium buffers and might play some important role in the control of the mutable connective tissue, which still needs to be defined and further investigated.

### Anti-calbindin 1 and anti-parvalbumin recognize the same antigen, echinoderms' calbindin-D32k

Bioinformatics analysis of members of the calbindin subfamily in the genome of the sea urchin *S. purpuratus* were performed to further single out which subfamily member is/are our markers recognizing. We focused on the presence of EF-hand domain-containing CBPs of the calbindin and parvalbumin subfamily, as these molecules are the ones recognized by these markers in vertebrates. Using this approach, we identified two proteins that are part of the calbindin subfamily of CBP, a putative calbindin 2 and secretagogin, but no proteins of the parvalbumin subfamily. Both of these proteins are predicted gene models that were formulated from the whole genome-sequencing project [17]. Thus we had two possibilities as to which antigen is/are the CBP marker recognizing in echinoderms.

Although the labeling of antigens by antibodies provides some information on the molecular structure of the antigen, in no way this provides definite proof of the antigen identity. Thus, additional studies were performed to better define the type(s) of CBP(s) of the calbindin subfamily being recognized by the antibodies in echinoderms. Western blot analyses to the three markers showed 32 kDa immunoreactive bands for anti-calbindin 1 and anti-parvalbumin. This observation, together with the similar labeling pattern observed in immunohistochemistry, raised the question as to whether these antibodies are recognizing the same antigen in *H. glaberrima*. This was certainly a possibility, especially considering that the location of fibers and cells labeled by immunohistochemistry is very similar for both. The location of the immunoreactivity within the immunopositive structures is the same, but the number of fibers and cells is not. This difference can be explained by a difference in the affinity of the antibodies for the antigen. Anti-calbindin 1 immunoreactivity was more intense than that of anti-parvalbumin or anti-calbindin 2, which is most likely the result of a higher affinity of the anti-calbindin 1 antibody for the antigen being recognized. Thus when taking these observations into account, in addition to the fact that no parvalbumin orthologue was identified in the *S. purpuratus* genome, the most plausible explanation for the parvalbumin immunoreactivity in the echinoderms is that this antibody is recognizing a conserved sequence of the EF-hand domain containing CBP in one of the calbindin subfamily members. We tested this hypothesis, and showed that both markers are recognizing a very similar, if not the same, antigen in radial nerve western blot. Furthermore, the *H. glaberrima* calbindin-D32k fragment western blots showed that the antigen in question is the orthologue of calbindin 2 in echinoderms, calbindin-D32k. These last sets of experiments show that indeed calbindin-D32k is present in the echinoderms and that it is the antigen being recognized by the anti-calbindin 1 and anti-parvalbumin markers used in our study. This last observation possibly holds true for the anti-calbindin 2 marker as well, though we only able to gather indirect evidence supporting this.

### Calbindin-D32k is present in Echinodermata

We have provided evidence of the presence of calbindin-D32k within the echinoderms, and this is the first report that establishes its presence in Echinodermata. Calbindin-D32k was also previously identified in *Drosophila* [13], although due to the lack of the full genome of the organism at the time, no evolutionary relation could be made with the members of the calbindin subfamily of EF-hand domain containing CBPs. The calbindin-D32k identified in *Drosophila* was named for its apparent molecular weight of 32 kDa in SDS-PAGE. Interestingly, calbindin-D32k and secretagogin are the only members of the calbindin subfamily present in invertebrates. Thus, our results also support the evolutionary conservation of the calbindin subfamily, which seems to have evolved from two primitive EF-hand domain-containing CBP. One of them gave rise to calbindin-D32k in invertebrates and to calbindin 1 and calbindin 2 in vertebrates, while the other gave rise to secretagogin in both, vertebrates and invertebrates. This hypothesis is also supported by findings from genomic sequencing of model organisms such as *D. melanogaster*, *S. purpuratus* or *S. kowalevskii*, in which only one ortholog of calbindin-D32k and one of secretagogin have been identified [17]; as well as previous works suggesting that calbindin 1 arose during the radiation of vertebrates and is a vertebrate-specific gene [13], [16], [19]. Nonetheless, there is still a need for a major pool of sequences from a wider variety of organisms to reach a better-informed conclusion, of when specifically did calbindin 1 arose. Thus we propose that the vertebrate calbindin 1 and calbindin 2 are co-orthologous of the calbindin-D32k of *Drosophila* and the calbindin-D32k of *S. purpuratus* and *H. glaberrima*.

In conclusion, our study contributes to a better understanding of the evolution of the calbindin subgroup of CBPs through the species and their role in the nervous system. We have identified a calbindin 2 ortholog in two echinoderms classes, Holothuroidea and Echinoidea, which is associated with the nervous system, more specifically with the connective tissue plexi. This protein corresponds to the calbindin-D32k previously identified in *Drosophila*, which is the ortholog of calbindin 2. Additionally, given the similarities found in sequence and structure, we show that the calbindin 1, parvalbumin, and calbindin 2 commercial antibodies are labeling the invertebrate ortholog of calbindin 2, calbindin-D32k in the echinoderm nervous system.
